# Production of functionally active *Penicillium chrysogenum *isopenicillin N synthase in the yeast *Hansenula polymorpha*

**DOI:** 10.1186/1472-6750-8-29

**Published:** 2008-03-19

**Authors:** Loknath Gidijala, Roel AL Bovenberg, Paul Klaassen, Ida J van der Klei, Marten Veenhuis, Jan AKW Kiel

**Affiliations:** 1 Molecular Cell Biology, Groningen Biomolecular Sciences and Biotechnology Institute (GBB), University of Groningen, P.O. Box 14, 9750 AA Haren, The Netherlands; 2 DSM Anti-Infectives, DSM Gist (624-0270), P.O. Box 425, 2600 AK, Delft, The Netherlands; 3 Kluyver Centre for Genomics of Industrial Fermentation, Julianalaan 67, 2628 BC Delft, The Netherlands

## Abstract

**Background:**

β-Lactams like penicillin and cephalosporin are among the oldest known antibiotics used against bacterial infections. Industrially, penicillin is produced by the filamentous fungus *Penicillium chrysogenum*. Our goal is to introduce the entire penicillin biosynthesis pathway into the methylotrophic yeast *Hansenula polymorpha*. Yeast species have the advantage of being versatile, easy to handle and cultivate, and possess superior fermentation properties relative to filamentous fungi. One of the fundamental challenges is to produce functionally active enzyme in *H. polymorpha*.

**Results:**

The *P. chrysogenum pcbC *gene encoding isopenicillin N synthase (IPNS) was successfully expressed in *H. polymorpha*, but the protein produced was unstable and inactive when the host was grown at its optimal growth temperature (37°C). Heterologously produced IPNS protein levels were enhanced when the cultivation temperature was lowered to either 25°C or 30°C. Furthermore, IPNS produced at these lower cultivation temperatures was functionally active. Localization experiments demonstrated that, like in *P. chrysogenum*, in *H. polymorpha *IPNS is located in the cytosol.

**Conclusion:**

In *P. chrysogenum*, the enzymes involved in penicillin production are compartmentalized in the cytosol and in microbodies. In this study, we focus on the cytosolic enzyme IPNS. Our data show that high amounts of functionally active IPNS enzyme can be produced in the heterologous host during cultivation at 25°C, the optimal growth temperature for *P. chrysogenum*. This is a new step forward in the metabolic reprogramming of *H. polymorpha *to produce penicillin.

## Background

β-lactam antibiotics like penicillins and cephalosporins belong to one of the largest-selling classes of drugs worldwide with a production of forty-five thousand tons in the year 2000 [1]. Penicillins and cephalosporins are produced by the filamentous fungi *Penicillium chrysogenum *and *Acremonium chrysogenum*, respectively, as well as some filamentous bacteria. These antibiotics possess as common structural motif the β-lactam ring [2]. Not surprisingly, the penicillin and cephalosporin biosynthetic pathways have the first two enzymatic steps in common. First, the non-ribosomal peptide synthetase δ(L-α-aminoadipyl)-L-cysteinyl-D-valine synthetase (ACVS) forms the tripeptide ACV. The formation of ACV acts as the committed step in both penicillin and cephalosporin biosynthesis. ACV is subsequently converted into isopenicillin N (IPN), which has the characteristic β-lactam backbone, by the enzyme isopenicillin N synthase (IPNS). Both ACVS and IPNS have been shown to be located in the cytosol in *P. chrysogenum *[3,4]. Subsequent replacement of the α-amino adipoyl side chain of IPN by the more hydrophobic phenylacetyl or phenoxyacetyl moieties occurs in *P. chrysogenum *in the specific environment of the microbody and results in the formation of penicillin [3,5]. On the other hand, epimerization of the α-amino adipoyl moiety followed by ring expansion leads to cephalosporin biosynthesis in *A. chrysogenum *[6]. So far, a requirement for specific organelles for cephalosporin production in this filamentous fungus is unknown.

Isopenicillin N synthase (IPNS) belongs to a class of non-heme ferrous iron dependent oxidoreductases. During its enzymatic reaction one molecule of oxygen is completely transformed into two water molecules by removal of four hydrogen atoms from the ACV tripeptide [7]. Detailed mechanistic studies of the IPNS enzyme were carried out using *Aspergillus nidulans *IPNS produced in *Escherichia coli *[8,9]. These studies showed that the formation of the β-lactam ring is carried out by an iron (IV)-oxy intermediate with the His212, Asp214, and His268 residues of *A. nidulans *IPNS forming the active center. Furthermore, a conserved Arg-Xxx-Ser oxidase motif is involved in binding of the ACV substrate [10].

Our objective is to introduce the penicillin biosynthesis pathway from *P. chrysogenum *into the methylotrophic yeast *Hansenula polymorpha*. Yeast species have the advantage of being versatile, easy to handle and cultivate, and possess superior fermentation properties relative to filamentous fungi. Additionally, introducing the penicillin biosynthesis pathway into this yeast species allows a better understanding of the function of microbodies in penicillin production. In the past, the microbody-localized proteins involved in penicillin biosynthesis in *P. chrysogenum *– isopenicillin N:acyl CoA acyltransferase (IAT) and phenylacetyl-CoA ligase (PCL) – were successfully produced in *H. polymorpha *in an active form [11,12]. Here we present our data on the expression of the *P. chrysogenum pcbC *gene encoding IPNS in *H. polymorpha*.

## Results and Discussion

### IPNS produced in *H. polymorpha *at 37°C is not stable

**Figure 1:**
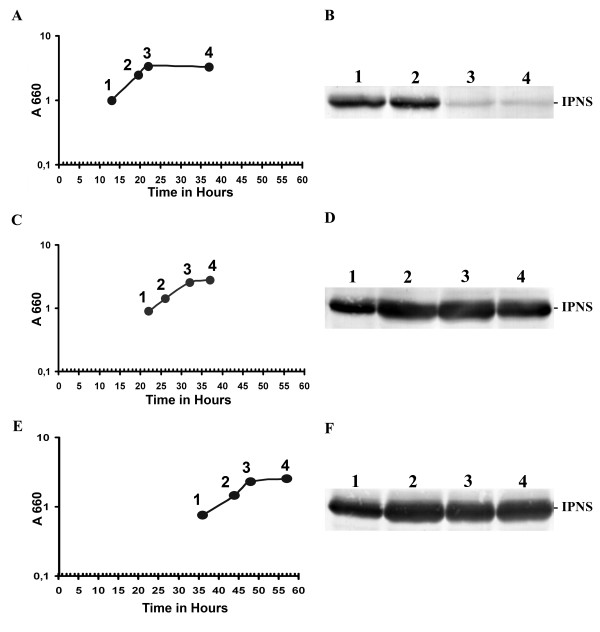
**Production of IPNS by *H. polymorpha *strain IPNS 4.2 cultivated at various temperatures**. Western blots showing IPNS production in cells of the *H. polymorpha *strain IPNS 4.2 cultivated at 37°C (panels A and B), 30°C (panels C and D) and 25°C (panels E and F). Aliquots were taken at different stages during cultivation at approximately equal optical densities (expressed as A_660 _and indicated as 1, 2, 3, and 4 in panels A, C and E). IPNS is produced in strain IPNS 4.2 at all growth temperatures. However, during later stages of growth relatively low IPNS protein levels were observed in cells grown at 37°C, while at 30°C and 25°C enhanced levels of IPNS protein were detected.

**Figure 2:**
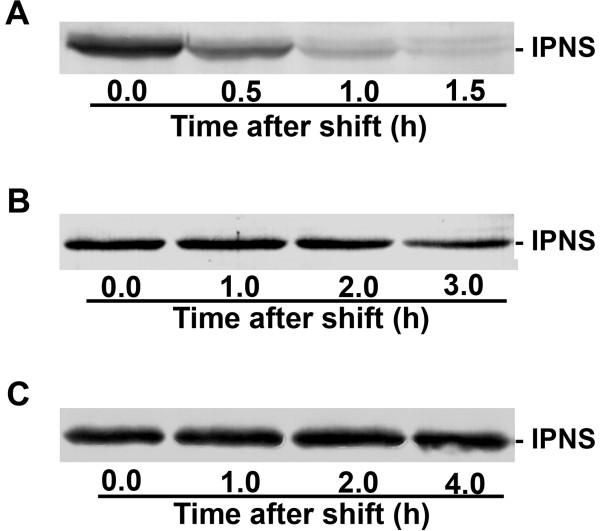
**Stability of IPNS produced by *H. polymorpha *strain IPNS 4.2 cultivated at various temperatures**. *H. polymorpha *IPNS 4.2 cells were cultivated on methanol media to the mid-exponential growth phase (A_660 _of approximately 1.5) at 37°C, 30°C and 25°C (panels A, B, and C, respectively). Subsequently, 0.5% glucose was added to repress the *AOX *promoter and stop further IPNS synthesis. Aliquots of the cultures were taken at the indicated time intervals after the shift. Western blots were prepared using equal volume fractions loaded per lane. For clarity, in panels B and C protein samples were diluted 10 times prior to loading. The results show that in IPNS 4.2 cells cultivated at 37°C IPNS protein is unstable since little protein is detected 1 h after the shift. In cells cultivated at lower temperatures, IPNS protein remains detectable at all time points.

The *P. chrysogenum pcbC *gene encoding IPNS was cloned downstream of the strong, inducible *H. polymorpha *alcohol oxidase promoter (P_*AOX*_) in pHIPX4 and was integrated at the P_*AOX *_locus in the *H. polymorpha *genome (see Methods). A strain, designated IPNS 4.2, containing the *pcbC *expression cassette was selected and cultivated at 37°C on methanol medium to induce the P_*AOX*_. Wild type *H. polymorpha *was used as control. To analyze the induction profile of IPNS protein, aliquots were taken at different stages of growth on methanol (Fig. 1, Panel A). Western blots were prepared using crude extracts of TCA precipitated cells and decorated with a polyclonal α-IPNS antiserum. This demonstrated (Fig. 1, Panel B) that strain IPNS 4.2 produces an α-IPNS specific protein of the expected size (approximately 37 kDa), that was absent in WT controls (data not shown). However, after the onset of the stationary growth phase IPNS levels declined rapidly (Fig. 1, Panel B). To check the stability of IPNS protein produced in *H. polymorpha*, cells of strain IPNS 4.2 were grown on methanol medium to mid-exponential growth phase, and then 0.5% glucose was added to the cultures, a treatment that results in complete repression of the P_*AOX*_. Subsequently, aliquots of equal volume were taken at regular time intervals and TCA precipitated. Western blot analysis showed that within 30 minutes after glucose addition the IPNS protein levels in the cells had decreased more than 50% (Fig. 2, Panel A), indicating that in *H. polymorpha *IPNS is not stable when produced at cultivation conditions physiological to the host.

### Effect of the cultivation temperature on IPNS production in *H. polymorpha*

Instability of heterologous produced protein in yeast can be attributed to improper folding in the unnatural host, or to absence of activation, both of which can result in turn-over of the protein [13]. Our assumption was that *P. chrysogenum *IPNS was not properly folding in *H. polymorpha*, which might be overcome by cultivating the recombinant strain at a reduced temperature. Since *P. chrysogenum *hyphae are usually grown at 25°C, we cultivated *H. polymorpha *strain IPNS 4.2 on methanol media at 30°C and 25°C again taking aliquots at different stages of growth (Fig. 1, Panels C and E). Under these conditions, the growth rate of the *H. polymorpha *cells is reduced. Western blots demonstrated that IPNS protein was present in *H. polymorpha *extracts at all stages of growth (Fig. 1, Panels D and F). We also analyzed the effect of the reduction in the growth temperature of strain IPNS 4.2 on the stability of IPNS. Cells grown at 30°C and 25°C on methanol media to the mid-exponential growth phase were shifted to 0.5% glucose in order to repress the P_*AOX *_and aliquots of equal volume were taken at regular time intervals after the shift. Western blot analysis demonstrated that IPNS protein was fully stable when produced at the lower growth temperatures (Fig. 2, Panels B and C).

**Figure 3:**
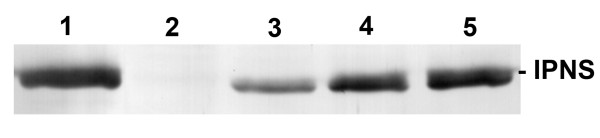
**Comparison of IPNS protein produced by *H. polymorpha *IPNS 4.2 cultivated at various temperatures**. Western blot showing the relative amounts of IPNS protein produced by mid-exponentially grown *H. polymorpha *IPNS 4.2 cells cultivated at 37°C, 30°C and 25°C (lanes 3, 4 and 5, respectively). As controls, *H. polymorpha *WT cells, cultivated on methanol at 25°C (lane 2), and *P. chrysogenum *cells, cultivated for 40 h on penicillin production media supplemented with phenylacetic acid (lane 1), are included. These demonstrate the specificity of the α-IPNS antiserum.

In order to directly compare the amount of IPNS protein produced in *H. polymorpha *IPNS 4.2 at the different cultivation temperatures, equal amounts of extracts from cells exponentially grown on methanol were analyzed by Western blotting. Extracts of identically grown *H. polymorpha *wild type cells and *P. chrysogenum *cells grown on penicillin production medium supplemented with phenylacetic acid were used as controls. This revealed that the steady state levels of IPNS protein produced in *H. polymorpha *at 25°C are significantly enhanced relative to production at 37°C and 30°C (Fig. 3). Additionally, the amount of IPNS produced at 25°C in the heterologous host is comparable to that produced in a high penicillin producing strain of *P. chrysogenum*.

### *H. polymorpha *IPNS 4.2 cells grown at reduced temperatures produce functionally active IPNS

**Figure 4:**
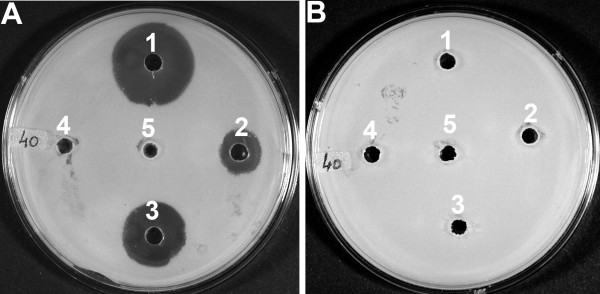
**IPNS produced by *H. polymorpha *strain IPNS 4.2 cultivated at 25°C is enzymatically active**. *H. polymorpha *IPNS 4.2 cells were cultivated on methanol-containing media to the mid-exponential growth phase at 37°C (well 4), 30°C (well 2) and 25°C (well 3). As controls, *H. polymorpha *WT cells, cultivated on methanol at 25°C (well 5), and *P. chrysogenum *cells, cultivated for 40 h on penicillin production media supplemented with phenylacetic acid (well 1) were used. Cell free extracts (40 μg of protein each) were incubated for 20 min at 25°C in a buffer containing ACV to allow production of IPN. Panel A: Reactions were terminated by methanol addition. After protein precipitation, an equal volume of reaction product was added to each well of a bioassay plate on which *M. luteus *had been plated as indicator strain. The results indicate that extracts prepared from IPNS 4.2 cells cultivated at 25°C and 30°C contain active IPNS enzyme, resulting in the formation of a halo. In contrast, extracts from IPNS 4.2 cells grown at 37°C show no activity. Panel B: As control, the reaction products were further incubated in the presence of penicillinase for an additional 30 min at 25°C. After that, the reaction mixtures were terminated by methanol addition and the reaction products analyzed on a bioassay plate as described in A. The absence of halos on the plate demonstrates that the IPNS 4.2 extracts indeed allow formation of the β-lactam ring.

We have determined the enzyme activity of IPNS produced in methanol grown cells of strain *H. polymorpha *IPNS 4.2 at different growth temperatures via a bioassay that utilizes the β-lactam sensitive bacterium *Micrococcus luteus *(ATCC 9341) as indicator strain [14]. Cells of the *H. polymorpha *host strain and *P. chrysogenum *served as controls. Crude extracts were prepared using glass beads as indicated in Methods and equal amounts of protein were incubated with substrate ACV. Subsequently, the resulting reaction product was used in the bioassay. When extracts prepared from the *H. polymorpha *strain IPNS 4.2 grown at 25°C and 30°C were used in this assay, this resulted in the formation of a zone of growth inhibition on the indicator plates (Fig. 4, Panel A). In contrast, extracts prepared from *H. polymorpha *WT and IPNS 4.2 grown at 37°C produced no inhibition zone. Also the use of higher amounts of extract from IPNS 4.2 cells grown at 37°C did not result in halo formation (data not shown). In order to check whether the halo formed by the reaction product of crude extracts of *H. polymorpha *IPNS 4.2 grown at 25°C and 30°C was caused by the synthesis of IPN, we added 50,000 IU of penicillinase to the reaction mixes and continued incubation at 25°C for an additional 30 minutes. The results (Fig. 4, Panel B) indicate that the product formed by the IPNS 4.2 extract is sensitive to penicillinase, confirming formation of the β-lactam structure. From this, we conclude that IPNS produced in *H. polymorpha *at 25°C and 30°C is functionally active.

### Localization of IPNS produced in *H. polymorpha*

**Figure 5:**
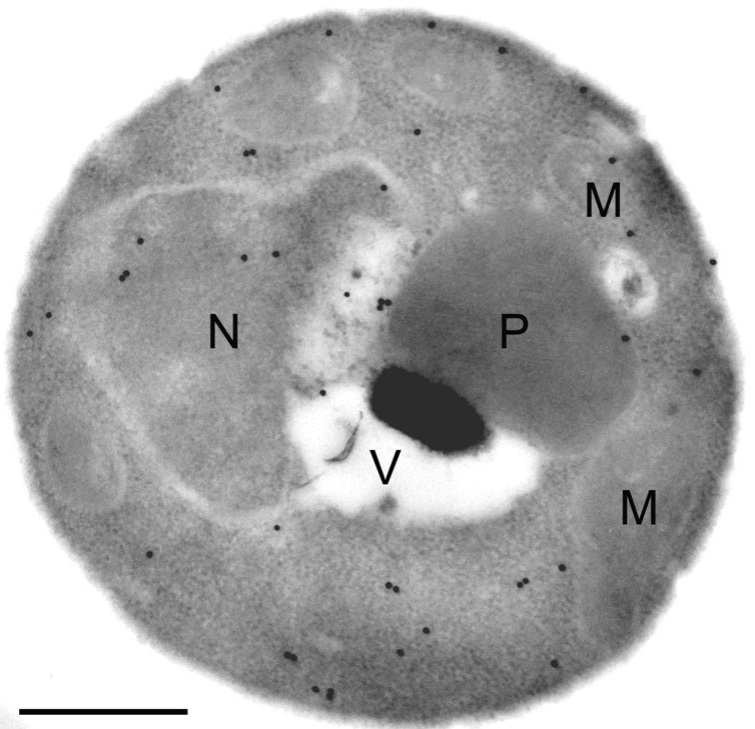
**Subcellular localization of IPNS protein produced in *H. polymorpha***. Methanol-grown *H. polymorpha *IPNS 4.2 cells were prepared for immunocytochemistry, using α-IPNS-specific antiserum. Gold particles are predominantly observed on the cytosol, but not on peroxisomes or mitochondria. Key: M, mitochondrion; N, nucleus; P, peroxisome; V, vacuole. The bar represents 0.5 μm.

In *P. chrysogenum *IPNS is a cytosolic protein [4]. Since two of the enzymes involved in penicillin biosynthesis (IAT and PCL) are located to microbodies in *P. chrysogenum *[4] [W.H. Meijer et al., unpublished results], it is important to know the subcellular location of IPNS in *H. polymorpha*. Cells of strain IPNS 4.2 were cultivated on methanol to induce IPNS production and prepared for immunocytochemistry using polyclonal α-IPNS antiserum. Immunogold labelling was only observed in the cytosol or on nuclear profiles (Fig. 5) as is characteristic for soluble proteins [15]. Microbodies and mitochondria invariably lacked specific labelling, which led us to conclude that IPNS produced in *H. polymorpha *is located in the cytosol.

## Conclusion

In this study, we demonstrate that *P. chrysogenum *IPNS, produced in the methylotrophic yeast *H. polymorpha*, is stable and functionally active when cells of the heterologous host are cultured at 25°C. In contrast, IPNS protein produced at 37°C, the ambient growth temperature of *H. polymorpha *is unstable and inactive. Our data support that the instability of IPNS at the higher temperature is caused by poor/slow folding of the protein. Cultivation of cells at the lower temperature is thought to slow down protein synthesis, which allows more time for proper folding. Indeed, the growth rate of *H. polymorpha *at 25°C is reduced as compared to 37°C (Fig. 1, compare Panels A and E). Structural analysis of *A. nidulans *IPNS [8] indicated that the protein contains β-sheets which form an active hydrophobic core that is shielded from the hydrophilic environment by α-helical structures. Previously, it was shown in *E. coli *that the solubility of IPNS improved significantly by lowering the cultivation temperature [16]. Thus, poor folding at high temperatures may be an intrinsic feature of IPNS.

Previously, we succeeded in producing IAT and PCL in *H. polymorpha *and to target these proteins to the correct subcellular location, the microbody [11,12]. Both proteins showed enzymatic activity in *H. polymorpha *cells cultured at 37°C, implying proper folding at the physiological growth temperature of the heterologous host. Nevertheless, since both proteins originate from *P. chrysogenum*, optimal enzyme activities are expected at 25°C. The observation that active IPNS is only produced in *H. polymorpha *cells cultured at 25°C implies that penicillin production in this heterologous host may only be successful at reduced growth temperatures.

Our next step on the way to insert the entire penicillin biosynthesis pathway into *H. polymorpha *will be the production of functionally active ACVS in the heterologous host. This undoubtedly is the most challenging step, since yeast genomes do not encode non-ribosomal peptide synthetases like ACVS, while in bacteria and filamentous fungi these proteins are widespread.

## Methods

### Microorganisms and growth conditions

*H. polymorpha *strains used are derivatives of NCYC495 *ade11.1 leu1.1 *[17] and were grown at 37°C, 30°C or 25°C in either (i) rich complex media (YPD) containing 1% yeast extract, 1% peptone and 1% glucose, (ii) selective media containing 0.67% yeast nitrogen base without amino acids (DIFCO) supplemented with 0.5% glucose (YND), or (iii) mineral medium (MM) as described by Van Dijken *et al*. [18], supplemented with 0.25% ammonium sulphate using 0.5% glucose or 0.5% methanol as carbon source. For growth on plates, 2% granulated agar was added to the media. Whenever necessary, media were supplemented with 30 μg/ml leucine and 20 μg/ml adenine. For biochemical analysis, selected strains were pre-cultured at least three times in MM containing glucose and subsequently shifted to MM containing methanol to induce expression of genes under the control of the P_*AOX*_.

A high penicillin producing *P. chrysogenum *strain (DSM anti-infectives, Delft, The Netherlands) was used as a control and was grown for 48 hours on a defined penicillin production medium supplemented with 0.05% phenylacetic acid [19].

For cloning purposes, *Escherichia coli *DH5α (Gibco-Brl, Gaithesburg, MD) was used and grown at 37°C in LB medium (1% bacto-tryptone, 0.5% yeast extract, 0.5% NaCl), supplemented with 50 μg/ml kanamycin when required.

### Miscellaneous DNA techniques

All DNA manipulations were carried out according to standard methods [20]. *H. polymorpha *cells were transformed by electroporation [21]. DNA modifying enzymes were used as recommended by the supplier (Roche, Almere, The Netherlands). *Pwo *polymerase was used for preparative polymerase chain reactions (PCR). Oligonucleotides were synthesized by Life Technologies (Breda, The Netherlands). DNA sequencing reactions were performed at BaseClear (Leiden, The Netherlands). For DNA sequence analysis, the Clone Manager 5 program (Scientific and Educational Software, Durham, USA) was used.

### Construction of *H. polymorpha *IPNS 4.2

For the construction of a *pcbC *expression plasmid a *Hind*III site was introduced at the 5' end and a *Sal*I site at the 3'end of *P. chrysogenum pcbC *by PCR with the primers ipns-F (5' *ACTAAGCTTATGGCTTCCACCCCCAAG *3') and ipns-R (5' *ATCGTCGACTCATGTCTGGCCGTTCTTGTTG *3') using DNA from a *P. chrysogenum *cDNA library as template [22]. The resulting 1002 bp DNA fragment was digested with *Hind*III and *Sal*I and cloned between the *Hind*III and *Sal*I sites of pHIPX4 [23] resulting in plasmid pHIPX4-pcbC. The nucleotide sequence of the cloned PCR fragment was confirmed by sequencing (data not shown). Subsequently, pHIPX4-pcbC was linearized with *Sph*I in the P_*AOX *_region and transformed into *H. polymorpha *NCYC495 *ade11.1 leu1.1 *cells. Fast growing leucine prototrophic transformants were selected, which are expected to carry multiple copies of the expression cassette [24]. Correct integration was confirmed by colony PCR using the genome-specific primer Aox-F (5' *TCACACCGTAACGCTTTATCGCC *3'), which corresponds to a region directly upstream of the *AOX *promoter and the plasmid-specific primer pcbC-R (5' *ATCTGGTCCTGGTGCTCCTTG *3'), which corresponds to the 5'end of the *pcbC *coding sequence. This will result in the formation of a product of 1898 bp exclusively in correct integrants (data not shown), one of which was designated IPNS 4.2.

### Biochemical methods

Crude extracts of *H. polymorpha *cells were prepared with glass beads basically as described previously [25]. Crude extracts of *P. chrysogenum *hyphae were prepared from 150 mg of lyophilized mycelium [26]. For Western blots, extracts of *H. polymorpha *and *P. chrysogenum *cells were prepared using the TCA method [27]. Protein concentrations were determined using the Bio-Rad Protein Assay system with bovine serum albumin as standard. SDS-PAGE and Western blotting were performed by established procedures. For Western blots *H. polymorpha *cells of the indicated strains were cultivated on methanol-containing media to induce the expression of the *AOX *promoter. At the indicated time intervals, aliquots of cells were taken and TCA precipitated. Extracts were prepared for Western blotting and decorated with polyclonal IPNS-specific antibodies [4]. Equal amounts of protein were loaded per lane, unless indicated otherwise.

### Bioassay for isopenicillin N synthase enzyme activity

Isopenicillin N synthase activity was determined in crude cell extracts prepared with extraction buffer (50 mM Tris-HCl, pH 8.0, 0.3 mM DTT, 1 mM PMSF) instead of phosphate buffer. Cell extracts corresponding to a fixed amount of protein (10 to 50 μg) were incubated at 25°C for 10 min in a final volume of 200 μl with 0.75 mM reduced Bis-ACV (Bachem Fein Chemicalien AG, Switzerland), 0.43 mM of FeSO_4_.7H_2_O as cofactor and 14.1 mM L-ascorbic acid as additive. After incubation, the reaction was terminated by addition of an equal volume of methanol to precipitate the proteins. After centrifugation, equal volume fractions of the supernatants were loaded in a well on an bioassay plate on which the indicator strain *Micrococcus luteus *ATCC 9341 had been plated. Plates were incubated overnight at 30°C. When penicillinase was used to degrade β-lactams in the reaction mixture, this was added (50,000 IU per reaction) prior to termination of the reaction with methanol.

### Morphological analysis

Intact cells were prepared for immunocytochemistry as described previously [25]. Ultrathin sections of unicryl-embedded cells were labeled using polyclonal antiserum raised in rabbit against IPNS and goat-anti-rabbit antibodies conjugated to gold according to the instruction of manufacturers (Amersham UK).

## Authors' contributions

LG participated in the experimental design, carried out the molecular genetic and biochemical experiments, participated in data interpretation and helped draft the manuscript. RALB, MV and IJvdK conceived the study. IJvdK participated in experimental design and data interpretation and critically read the manuscript.

MV participated in the experimental design, was involved in electron microscopy, participated in data interpretation and helped draft the manuscript. PK participated in IPNS activity measurements. JAKWK directly supervised the project, participated in its experimental design and data interpretation and was responsible for writing the manuscript. All authors have read and approved the manuscript.
